# Drug target assessments: classifying target modulation and associated health effects using multi-level BERT-based classification models

**DOI:** 10.1093/bioadv/vbaf043

**Published:** 2025-03-08

**Authors:** Jennifer Venhorst, Gino Kalkman

**Affiliations:** Biomedical and Digital Health, The Netherlands Organization for Applied Scientific Research (TNO), Utrecht 3584 CB, The Netherlands; Biomedical and Digital Health, The Netherlands Organization for Applied Scientific Research (TNO), Utrecht 3584 CB, The Netherlands

## Abstract

**Motivation:**

Drug target selection determines the success of the drug development pipeline. Therefore, novel drug targets need to be assessed for their therapeutic benefits/risks at the earliest stage possible. Where manual risk/benefit analyses are often user-biased and time-consuming, Large Language Models can offer a systematic and efficient approach to curating and analysing literature. Currently, publicly available Large Language Models are lacking for this task, while public platforms for target assessments are limited to co-occurrences.

**Results:**

BERT-models for multi-level classification of drug target–health effect relationships described in PubMed were developed. Relationships were classified based on (i) causality; (ii) direction of target modulation; (iii) direction of the associated health effect. The models showed competitive performances with F1 scores between 0.86 and 0.92 and their applicability was demonstrated using ADAM33 and OSM as case study. The developed classification pipeline is the first to allow detailed classification of drug target–health effect relationships. The models provide mechanistic insight into how target modulation affects health and disease, both from an efficacy and safety perspective. The models, deployed on the whole of PubMed and available through the TargetTri platform, are expected to offer a significant advancement in artificial intelligence-assisted target identification and evaluation.

**Availability and implementation:**

https://www.targettri.com.

## 1 Introduction

**Figure 1.**
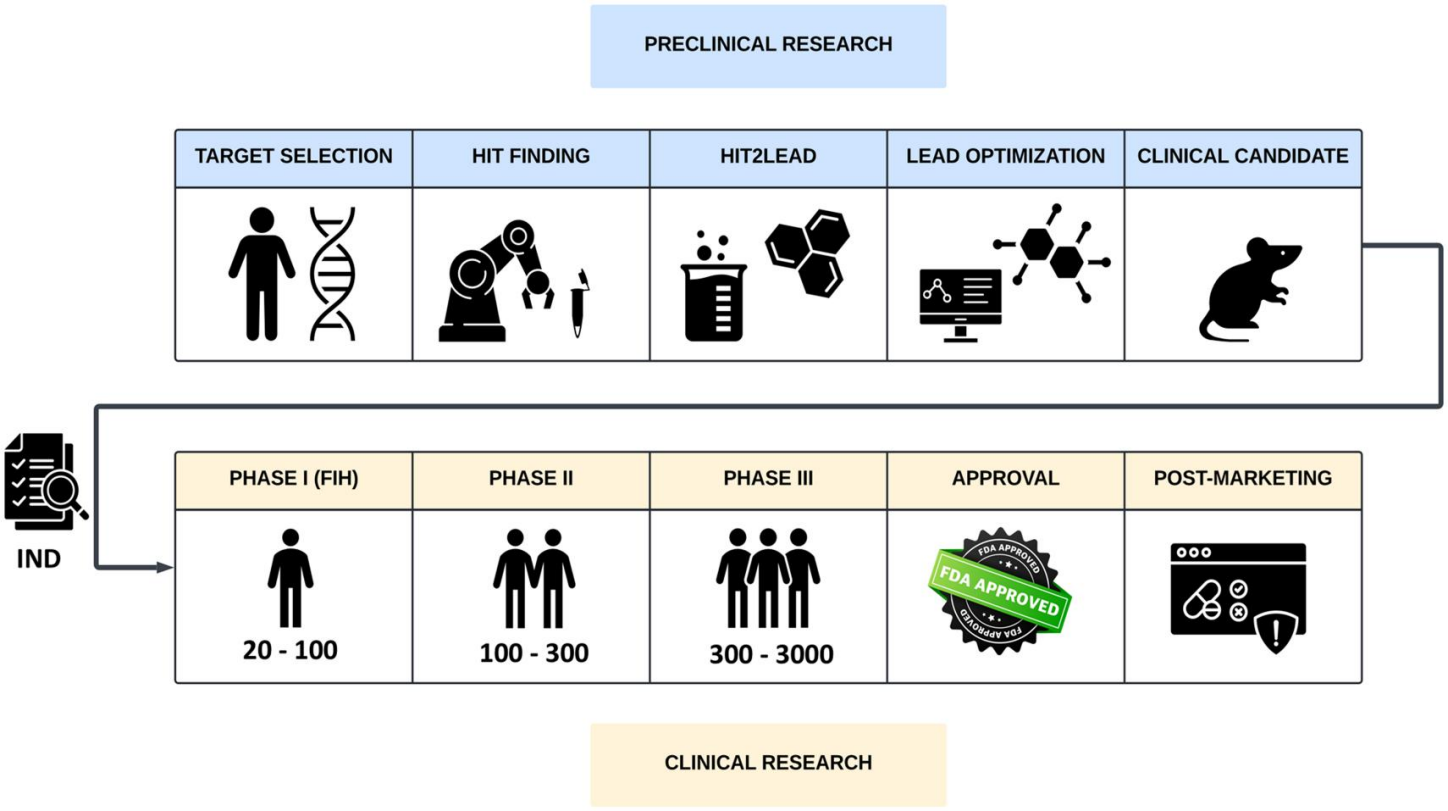
Schematic representation of the drug development pipeline. Frontloading of target assessments (target selection phase) has reduced attrition rates. During these assessments, perturbations of the target’s function in the disease are investigated to mechanistically understand how a human protein—and potential drug target—contributes to a disease process. These insights indicate how the protein can be influenced to reverse or halt disease. Similarly, once a therapeutic strategy has been established (e.g. inhibition of the protein serving as drug target), potential adverse effects of this therapy can be investigated using drug target assessments.

Drug discovery, which starts with drug target identification and evaluation, is both lengthy and costly with limited chance of success. Currently, the clinical success rate of a drug from candidate to market is only 10%–20% (Sun *et al.* 2022). The primary source of failure in drug development is a lack of demonstrated efficacy, followed by toxicological observations. Often on-target effects are at the core of these failures (Cook *et al.* 2014). In particular, a lack of mechanistic understanding of the target’s role in health and disease underlies efficacy-based failures. In accordance, the odds of approval are lowest for drugs with a new mechanism of action against a previously ‘undrugged’ target protein, and for diseases with an ill-understood pathogenesis (Fogel 2018, Hingorani *et al.* 2019, Yamaguchi *et al.* 2021). Nevertheless, attrition rates have decreased during the last decade. This has partly been attributed to the emphasis on mechanistically evaluating and validating potential drug targets in preclinical research with target assessments now being performed at the very beginning of the development pipeline (Fig. 1) (Pammolli *et al.* 2020). The importance of unbiased, data-driven decisions for target selection has recently been illustrated by Pfizer, stressing that a deep understanding of the biology of disease and the target’s mechanistic role is a crucial factor for success (Fernando *et al.* 2022). This is where AI-assisted target assessments can significantly contribute to the reduction of drug attrition due to on-target effects, both in terms of safety and efficacy.

Drug target assessments are generally performed by experts who are faced with a number of hurdles. These hurdles include the enormous volume of (often unstructured) data, most notably the literature, which needs to be analysed. Although strategies have been developed to support target assessments (Venhorst *et al.* 2019, Emmerich *et al.* 2021), these in themselves do not reduce the amount of data that needs to be reviewed, annotated for relevance, and processed. In addition, expert-based assessments are prone to personal bias. To leverage publicly available data in understanding successful drug targets and their downstream effects, whilst overcoming these hurdles, the application of artificial intelligence (AI) is taking a firm hold in the drug discovery process (Brown *et al.* 2018, Bender and Cortés-Ciriano 2021). Text-mining strategies, such as Bidirectional Encoder Representations from Transformers (BERT) models, can reduce noise and limit information to a quantity that can be humanly processed in a reasonable time frame when combined with hierarchical taxonomies (Yang *et al.* 2022).

Recently, pre-trained text-mining models like BERT (Devlin *et al.* 2019) have been shown to improve in performance when trained on domain-specific data. Fine-tuning can further optimize text-mining tasks as, e.g. shown for a BERT-based model pertaining to drug–target relationships (Aldahdooh *et al.* 2022). Other prediction models described include drug–drug (Liu *et al.* 2016) and drug—adverse effect relationships (Xu and Wang 2014). However, agnostic models that can classify all drug target–health effects on a detailed level are currently lacking in the public domain. Therefore, decision support specifically focusing on drug target assessments using text-mining strategies is currently not available.

**Figure 2.**
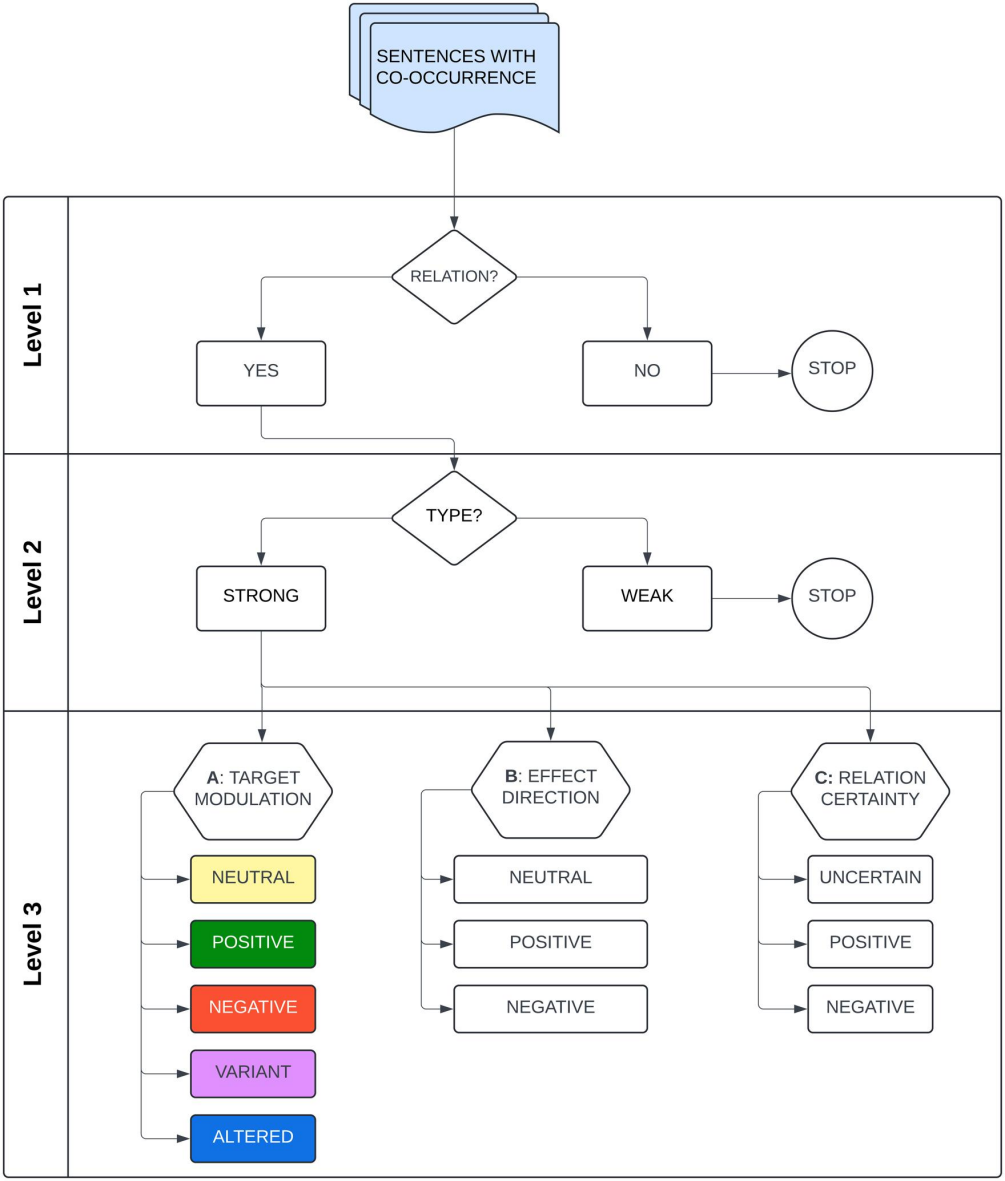
Classification scheme used for sentences containing a drug target and a health effect term. The first level determines whether the sentence indeed contains a relationship and does not constitute a simple co-occurrence. When classified as ‘YES’, the relation is subsequently classified as ‘WEAK’ or ‘STRONG’ on level 2. ‘WEAK’ relations describe indirect relationships such as contextual information or a study aim; ‘STRONG’ relations encompass associations and causal relationships between the drug target and health effect (Supplementary Tables S1.1–S1.5). If the relation is classified as a co-occurrence (classification level 1; class ‘NO’), the other levels are disregarded. This is also true for ‘WEAK’ relationships (classification level 2). Relations classified as ‘STRONG’ are further classified with respect to drug target/health effect modulation (classification level 3A/B) and the certainty of the described relationship (classification level 3C).

In this study, we developed a pipeline that performs multi-level classification of drug target–health effect relations for those features that are deemed crucial for drug target assessments (Fig. 2). For training and validation, approximately 4000 sentences were manually annotated by multiple experts. The resulting BERT-based models first classify whether a sentence containing the two entities represents a co-occurrence, an indirect, or a direct relationship. Subsequently, the direction of both drug target modulation and the associated health effect are assessed for direct relationships. In addition, the certainty of the described relationship is classified. A robust performance was observed for both the training and validation set. To illustrate the applicability of the models in a real-world example, we analysed the health effects of target modulation using OSM (Oncostatin M) and ADAM33 (a disintegrin and metalloproteinase domain 33) in the context of target identification and target safety assessments. The outcome of the classification models can be used to support target selection within a specific disease context, or for designing target derisking strategies. To allow assessment of any potential drug target, the BERT-based models have been deployed to the 24 million PubMed abstracts available in the TargetTri platform (https://www.targettri.com) which contains all human reviewed proteins from UniProt. The models are expected to significantly facilitate target discovery and evaluation based on mechanistic insights regarding their biological role.

## 2 Methods

### 2.1 Definition of drug targets and health effects

Any human protein, in theory, can constitute a potential drug target given that such a protein performs a physiological role which can be perturbed, resulting in disease. Therefore, in this study, a drug target is defined as any characterized and reviewed human protein described in the UniProt database. It should be noted that—by using this definition—the term ‘drug target’ primarily relates to the protein’s (causal) role in disease. To progress such a drug target in the drug development process, additional criteria need to be met. This includes, e.g. being amenable to interventions (druggability), availability of mechanistic and functional screening assays, and lack of on-target toxicities (Venhorst *et al.* 2019). Health effects are defined throughout this paper as any effect or process (molecular or biological) that is relevant for the initiation, progression, halting, or reversal of disease, or for the maintenance or promotion of health. As a result of these definitions, all protein–health effect pairs in a sentence are classified.

### 2.2 Data generation for training/external validation

Using the protein and health effect ontologies, and the algorithms described in the Supplementary Material (Supplementary S1), a data-processing pipeline was implemented that identifies all drug targets (protein/gene) and health effects (diseases, physiology, adverse effects, biological processes) described in the title and abstract of PubMed articles published from the year 2000 onwards. PubMed data is processed with this pipeline on a daily basis and stored in a dedicated ElasticSearch database. From this database, we queried over 3000 sentences from titles and abstracts of PubMed articles published in 2023 containing at least a co-occurrence of a protein and health effect. To ensure a balanced training and validation set, with sufficient representation of each classification level and class, the datasets were manually enriched using the TargetTri interface and download options, using arbitrary proteins. For example to select sentences with positive target modulation, the filtering option on stimulatory mechanism of action available in TargetTri was used. This filtering option matches keywords pertaining to target stimulation such as ‘agonist’ and ‘positive allosteric modulator’. Other filters that were used were e.g. those regarding inhibitory modes of actions, transgenic studies, and drug modalities. All sentences were subsequently classified independently by multiple annotators.

### 2.3 Annotations

Prior to performing the annotations, a classification scheme with ground rules was defined. This scheme was based on our experience with target evaluations and the information needed to assess mechanism-based benefits and risks of target modulation. A first set of ∼800 sentences was annotated by four experts with a background in the biomedical and linguistic domain in batches of about 100 sentences. Based on disagreements between the annotators, rules were expanded and further specified. This included, e.g. the expansion of verbs describing an increase and decrease of the health effects. Associations between a drug target and health effect, initially classified as ‘weak’, were annotated as ‘strong’ (level 2) in later annotation rounds, because such relationships provide valuable information for target assessments. It also needed to be agreed upon that a positive correlation between target and health effect did not infer an increase of the health effect. Rather, it simply indicates that the target and health effect follow the same directional pattern and therefore should be classified as ‘neutral’. Furthermore, the first batch of sentences highlighted the need for additional classification categories, resulting in the final classification scheme illustrated in Fig. 2. For example, the description of a strong relationship (classification levels 2) did not provide insight as to whether this relationship was affirmative (‘positive’), negated (‘negative’), or uncertain. Therefore, a new classification level (3C) was introduced. Once consensus on the definitions of the annotation rules had been reached, another batch of sentences was annotated by at least two experts, resulting in a final training set of 3477 sentences. For the second batch, inter-annotator agreement (IAA) scores were calculated for each of the classification levels using the Cohen Kappa Coefficient. Initial inter-annotator agreement (IAA) scores ranged from 0.58 to 0.7 and thus required the annotators to further clarify the set of annotation rules for sentences that were still interpreted ambiguously, aiming for a more consistent analysis of specific verbs and expressions. For instance, sentences stating that target X plays a crucial role in effect Y from then on were consistently classified into the ‘neutral’ health effect direction category, as they require an analysis of the wider context to define how exactly the target modifies the effect. Based on decisions like this, the annotators reconsidered their work resulting in IAA scores between 0.74 and 0.88, with most of the remaining disagreements being attributable to accidental mistakes or ambiguous sentences. In addition to the training set, an external validation set consisting of 414 sentences was generated and annotated using the same methodology as for the training set.

### 2.4 Classification scheme

The classification scheme applied by the annotators encompasses three levels. The first binary classification level (level 1 in Fig. 2) distinguishes co-occurrences from sentences describing a relationship between a protein and health effect. The second binary classification level (level 2 in Fig. 2) distinguishes weak (indirect) relationships from strong (direct) ones. An example of a weak relationship is one that describes the context in which a certain target-related observation is made (see also Supplementary Table S1.2). In, e.g. the sentence ‘Angiopoietin-2 (ANGPT2) is reported to facilitate angiogenesis, …. in various cancers, …’ this context is cancer. Therefore, the relationship between ANGPT2 and cancers is classified as weak. Strong relations (Supplementary Table S1.2) describe either an association or a causal relationship between a drug target and a health effect. On classification level 3 (Fig. 2), strong relations are further specified with respect to the direction of target modulation (e.g. activation or inhibition; level 3A) and the direction of the health effect (e.g. increase or decrease; level 3B) as well as the certainty of the relationship (level 3C).

In terms of level 3A, explicit modulation implying an activation or inactivation of the target is captured by the respective classes ‘positive’ and ‘negative’. When no explicit modulation is mentioned, the class is set to ‘neutral’. During the iterative annotation process the need for additional granularity surfaced to capture those cases where the protein target is modified with unknown effect on its activity. This resulted in the classes ‘variant’—for genetic variants without further specification—and ‘altered’. The latter represents a collection of cases, including e.g. cleavage and protein binding events. Similar to level 3A, the direction of the health effect is classified on level 3B as ‘positive’ (increase), ‘negative’ (decrease) and ‘neutral’ (no explicit direction of modulation reported).

All classification levels, classes and annotation rules are exemplified in Supplementary Tables S1.1–S1.5. The distribution of the classes per classification level is listed in Supplementary Tables S1.6 and S1.7.

### 2.5 BERT-model fine-tuning

BERT is a language representation model using masked language models and ‘next sentence prediction’ to generate pretrained bidirectional representations (Devlin *et al.* 2019). BERT models can be finetuned for a specific task using labelled data to improve performance.

These models use masking of entities to solve the out-of-vocabulary (OOV) problem, which typically plays a role in tasks like entity recognition and relation classification (Devlin *et al.* 2019). OOV problems occur when the presence of named entities that are not part of the lexicon of the model negatively impact its performance. This can be overcome by replacing entities with the [MASK] token, forcing a model to focus more on the semantic context of an entity than on the information conveyed in the entity itself. During training of the BERT-based models, the effect of masking was investigated on the performance. The [PROTEIN] and [EFFECT] masking tokens were used.

Hyperparameter optimization was performed to attain optimal performance. We took the default hyperparameter configuration for text classification models as our starting point (Hugging Face 2024, ‘Text Classification’). Subsequently, we used GridSearch to explore the hyperparameter space, evaluating different combinations of parameters based on the F1 score metric. The optimal set of hyperparameters is listed in Supplementary Table S2.1.

### 2.6 Model performance

Three BERT-based models were finetuned on our relation classification tasks and their performance was determined. The models were selected based on their pretraining data in order to determine the impact of domain-specific training on the ability to correctly classify drug target–health effects. These models were: (i) BERT base—pretrained exclusively on general-domain literature (3.3 billion words from Wikipedia and Books); (ii) PubMedBert (Gu *et al.* 2022)—pretrained exclusively on biomedical-domain literature (3.1 billion words from PubMed); and (iii) BioBERT (J. Lee *et al.* 2020)—initialized with the pretrained weights from the BERT base model, then further pretrained on biomedical-domain literature (4.5 billion words from PubMed, 13.5 billion words from PMC).

The performance of the fine-tuned BERT models was assessed using the following metrics: accuracy, precision, recall (also sensitivity or true positive rate), and F1 score (Supplementary Table S2.2). The F1 score is the harmonic mean of precision and recall and, as such, offers a balanced assessment of the model's performance. This is of particular importance in scenarios where class imbalance exists, as is the case in our dataset (Supplementary Table S1.6). For all classification levels, macro-averaging was performed for the precision and recall metrics, i.e. these values were averaged across all classes to get the final macro-averaged precision, recall and F1 scores.

For the training set, a stratified K-fold cross-validation technique was used combined with data shuffling strategies. For this, we utilized the StratifiedKFold class implemented in the scikit-learn library (Pedregosa *et al.* 2011). This class randomly partitions the dataset into K subsets while preserving the class distribution, thereby allowing for a more dependable estimate of the model's performance across diverse data distributions. Based on experiments, a 5-fold cross-validation was performed using 80% of the data for training and 20% for performance evaluation. By applying stratified partitioning methods, the folds were guaranteed to have the same class distribution as the original dataset. The final set of BERT models were generated using the complete training set, which was followed by a performance evaluation of the models based on the external validation set.

### 2.7 Case study

All sentences stored in the TargetTri ElasticSearch database (Supplementary S1) containing one of the protein/gene names denoting either ADAM33 (A Disintegrin And Metalloproteinase domain 33) or OSM (Oncostatin M) were extracted. Subsequently, the relation classification pipeline was deployed on these sentences. Results from text-mining were compared to data available in the following databases: UniProt (www.uniprot.org), CTD (https://ctdbase.org/), ClinVar (https://www.ncbi.nlm.nih.gov/clinvar/), GWAS catalogue (https://www.ebi.ac.uk/gwas/), and OMIM (https://www.omim.org/).

## 3 Results

### 3.1 Model performance training set

The performance of our finetuned models was assessed in terms of accuracy, precision, recall, and F1 score. Table 1 lists the mean and standard deviation (SD) of these metrics across the five folds of cross-validation performed for each of the classification levels in the training set. Comparative analyses between the three types of BERT-based models revealed that BioBERT yielded the highest performance scores for most of the five classification levels, closely followed by PubMedBERT (Table 1). For level 3A, both models yielded identical F1 scores, while PubMedBERT slightly outperformed BioBERT on level 3B.

**Table 1.** Macro-average performance of the classification models (Fig. 2) for the training set (mean ± SD across five folds).

Classification level	Model	Accuracy	Precision	Recall	F1
1	Generic BERT	0.88 ± 0.014	0.87 ± 0.024	0.83 ± 0.015	0.84 ± 0.017
	PubMedBERT	0.94 ± 0.006	0.93 ± 0.008	0.91 ± 0.009	0.92 ± 0.008
	**BioBERT**	0.95 ± 0.01	0.95 ± 0.012	0.92 ± 0.016	**0.93 ± 0.014**
2	Generic BERT	0.95 ± 0.002	0.92 ± 0.011	0.92 ± 0.009	0.92 ± 0.003
	PubMedBERT	0.95 ± 0.010	0.93 ± 0.013	0.92 ± 0.017	0.92 ± 0.015
	**BioBERT**	0.96 ± 0.007	0.94 ± 0.01	0.93 ± 0.013	**0.94 ± 0.011**
3A	Generic BERT	0.87 ± 0.015	0.85 ± 0.011	0.84 ± 0.01	0.85 ± 0.01
	**PubMedBERT**	0.92 ± 0.013	0.92 ± 0.016	0.91 ± 0.017	**0.91 ± 0.016**
	BioBERT	0.92 ± 0.011	0.91 ± 0.012	0.9 ± 0.016	0.9 ± 0.013
3B	Generic BERT	0.89 ± 0.014	0.88 ± 0.015	0.88 ± 0.013	0.88 ± 0.014
	PubMedBERT	0.91 ± 0.014	0.91 ± 0.014	0.91 ± 0.013	0.91 ± 0.014
	**BioBERT**	0.91 ± 0.005	0.91 ± 0.004	0.91 ± 0.005	**0.91 ± 0.004**
3C	Generic BERT	0.94 ± 0.008	0.87 ± 0.015	0.91 ± 0.012	0.89 ± 0.008
	PubMedBERT	0.96 ± 0.005	0.91 ± 0.013	0.92 ± 0.012	0.92 ± 0.005
	**BioBERT**	0.97 ± 0.007	0.93 ± 0.019	0.94 ± 0.018	**0.93 ± 0.008**

Bold values show the best performance in F1 score.

The generic BERT model, though still achieving an average F1 score of 0.84, scored significantly lower for most classification levels (Table 1). Especially for levels 1 and 3A generic BERT was outperformed by BioBERT. Therefore, it can be concluded that the BERT architecture offers a useful basis for the task of relation classification, because it accounts for bidirectional contextual patterns and provides rich representations of semantic features. However, using a domain-specific base model that is further pretrained on large volumes of domain-specific texts significantly improves the performance of the BERT modelling architecture.

The best overall performance was obtained with BioBERT. The BioBERT-based models achieved an average accuracy between 0.91 and 0.97, a macro-average precision between 0.91 and 0.95, a macro-average recall between 0.9 and 0.94, and a macro-average F1-score between 0.9 and 0.94. As such, further studies were performed with these models. Performance metrics for the individual classes are listed in Supplementary Tables S2.3–S2.7. The best performance was obtained with masking for all classification levels except 3A (target modulation) and 3B (direction of effect). For these levels masking was not incorporated in the final model. The higher performance without masking can be explained by the fact that the entities may contain information that is necessary to assign the correct classes at these levels. For example, the drug target entity ‘SIRT1-KO’ indicates the class ‘decrease’ for target modulation. With masking, this information would be lost, resulting in an incorrect classification.

### 3.2 Model performance test set

As a last step, the five final BioBERT classification models were used to predict the classes of the external validation set consisting of 414 sentences. Table 2 lists a summary of the evaluation metrics for each of the models, indicating per metric the mean score for all classes. A detailed overview of the evaluation metrics per class can be found in Supplementary Tables S2.8–S2.12. A comparison between the macro-average performance of BioBERT and that of the other BERT-based models on the external validation set is provided in Supplementary Table S2.13. The results show that the classification models achieve good performance on the identification and classification of relationships between drug targets and health effects on all classification levels.

**Table 2.** Macro-average performance of the BioBERT-based classification models for the external validation set.

Classification level	Accuracy	Precision	Recall	F1
1	0.95	0.93	0.92	0.92
2	0.90	0.87	0.85	0.86
3A	0.89	0.88	0.88	0.88
3B	0.88	0.87	0.88	0.88
3C	0.98	0.96	0.96	0.96

An imbalance in the distribution of classes within classification levels was observed to varying extent, despite dedicated efforts to populate less represented classes (Supplementary Table S1.6). This imbalance appears inherent to manual annotations using (semi-)random sentence selections, as it is likely a reflection of the data incorporated in PubMed. Certain types of data, e.g. negative observations or protein modifications, are less often described in the literature. As evidenced by the results obtained for the validation set, model performance appeared robust despite class imbalance. This may in part be due to more limited contextual variation for, e.g. negative relations, making classification a less complex task.

Based on the overall good performance of the BioBERT-based classification models, these models were integrated in our TargetTri platform.

### 3.3 Case studies

To demonstrate the applicability of the BERT-based models for target assessment, we selected and benchmarked two case studies.

#### 3.3.1 A disintegrin and metalloproteinase domain 33

**Figure 3.**
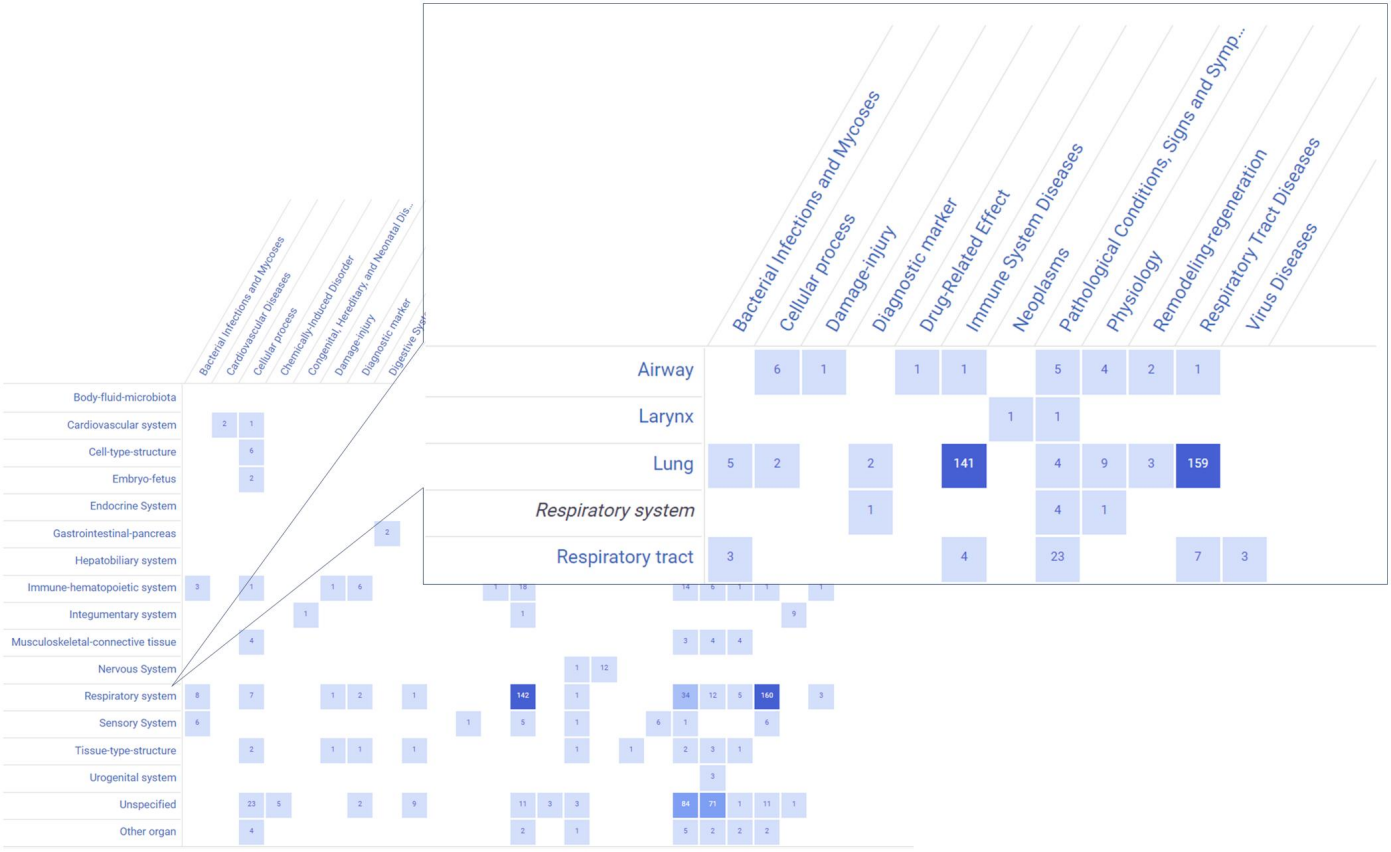
TargetTri results obtained for sentences containing ADAM33 co-occurrences with a health effect, structured by health effect (columns), and organ system (rows). The values in the heatmap indicate the number of PubMed publications found for the respective intersections and show that the majority of health effects occurs in the respiratory system and pertains to immune system or respiratory tract diseases.

To assess whether the BERT-based models can support target identification, we benchmarked the classification of ADAM33 against curated database information in terms of potential therapeutic benefits. ADAM33 belongs to the family of matrix metalloproteinases (MMPs). MMPs are important for regulating the turnover of extracellular matrix (ECM) components and have been implicated in various diseases, such as cancer (Mustafa *et al.* 2022) and vision-related diseases (Drankowska *et al.* 2019). Extraction from our database of all sentences containing ADAM33 in co-occurrence with a health effect resulted in 216 unique papers, with the majority of health effects occurring in the respiratory system and pertaining to immune system or respiratory tract diseases (Fig. 3). The low number of papers indicate that ADAM33 is an exploratory target.

**Figure 4.**
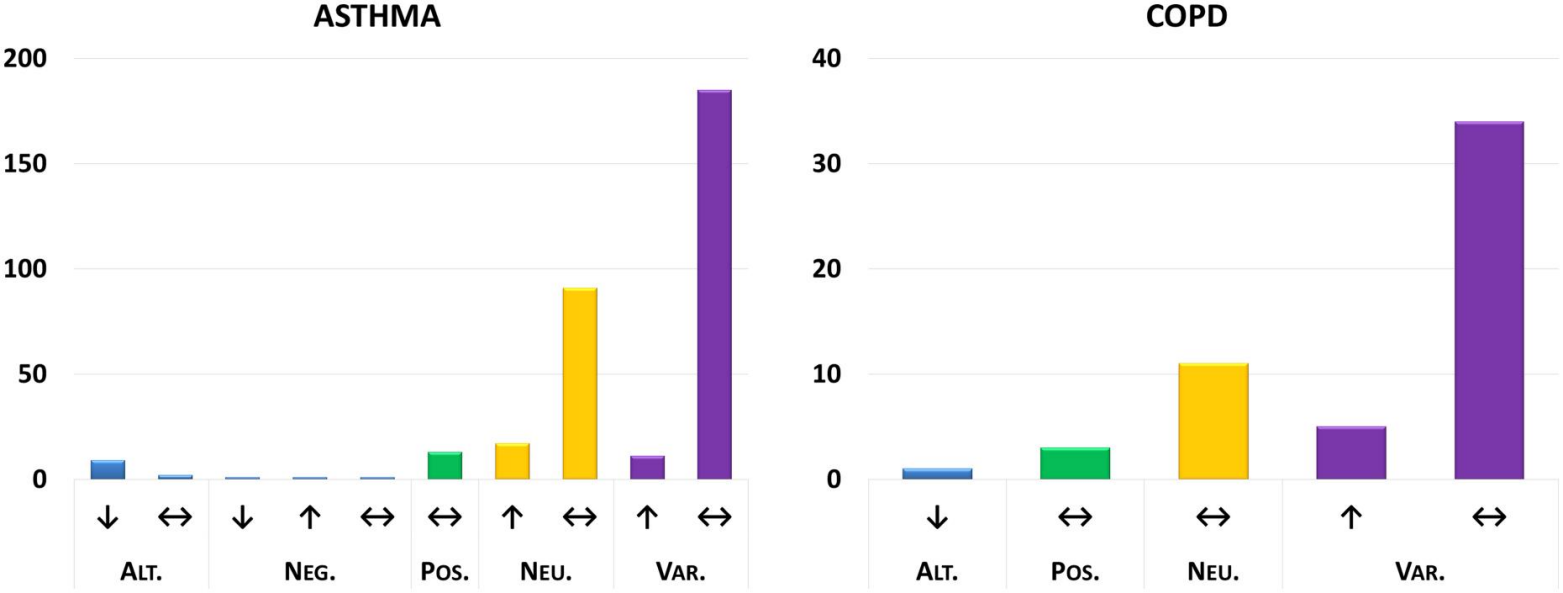
Classification of the top-ranked health effects showing that genetic variations [Variant (Var.)] of this protein are associated (Neutral) with asthma and COPD. Target modulation is indicated by color and text (Alt.; Neg.; Pos.; Neu.; Var.); the direction of the health effect is indicated by arrows. The *y*-axis shows the number of classified sentences.

Deployment of our classification pipeline showed that the top five most occurring effects having a strong relation (classification level 2) with ADAM33 were asthma, chronic obstructive pulmonary disease (COPD), lung function, allergic rhinitis, and airway remodelling. The top ranked health effect not occurring in the respiratory tract was psoriasis. When applying majority voting, it is clear from Fig. 4 and Supplementary Fig. S3.1 that the top ranked health effects of ADAM33 are primarily limited to associations (class level 3B: neutral) with (genetic variants of) the gene/protein (class level 3A: variant or neutral). Target–disease linkage evidenced by genetic variants (polymorphisms), especially when supported by multiple independent investigations, are often a starting point for further exploration of a protein/gene as potential drug target (Floris *et al.* 2018, Emmerich *et al.* 2021). Therefore, although the genetic variants do not indicate a clear mode of action for therapy, the classification results do warrant further exploration of ADAM33 as potential target for Asthma or COPD.

The associations observed with our models were benchmarked against information available in public databases. In these databases, curated relationships with ADAM33 included Asthma (UniProt, CTD and OMIM) and breast cancer (CTD). The latter was also identified by our classification pipeline (Supplementary Table S3.1). In accordance with our classification models, the databases only report of an association between ADAM33 and the respective effect, without an explicit mode of action. The associations (class level 3A: neutral) with COPD, allergic rhinitis, psoriasis, and airway remodelling were not found in the databases queried. This demonstrates the ability of our text-mining pipeline to uncover insights regarding target modulation and the direction of effects that cannot be readily extracted from other sources. For psoriasis, negative associations for ADAM33 were also retrieved (Supplementary Table S3.1), indicating potential disagreements between studies. Such insights are highly valuable for hypothesis substantiation and decision making in target selection.

#### 3.3.2 Oncostatin M

**Figure 5.**
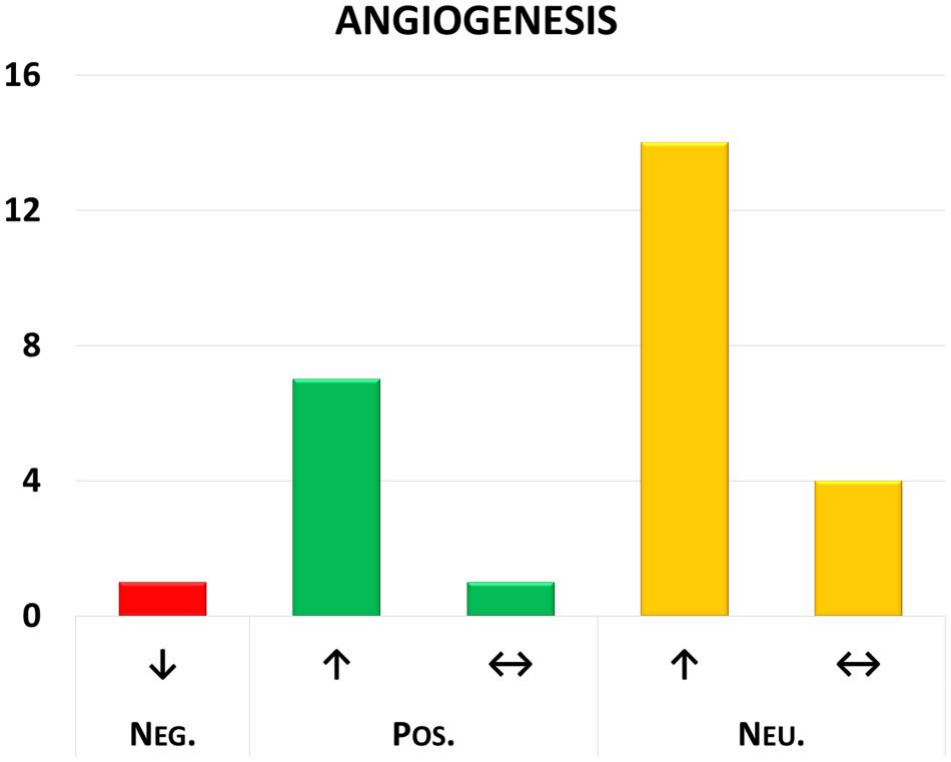
Classification of strong relations of OSM with angiogenesis showing that (activation of) OSM increases angiogenesis, and that its inhibition reduces angiogenesis. Target modulation is indicated by color and text (Neg.; Pos.; Neu.); the direction of the health effect is indicated by arrows. The *y*-axis shows the number of classified sentences.

Besides target identification, classification of target–health effects can be used to assess safety liabilities of interventions. This application was benchmarked using OSM as case study. OSM belongs to the interleukin-6 (IL-6) cytokine family and is a secreted cytokine of 252 amino acids. Members of the IL-6 family play important roles in diseases, such as chronic inflammation, autoimmunity, infectious diseases, and cancer. As this protein has previously been subjected to an extensive risk/benefit analysis in the context of atherosclerosis treatment (Rankouhi *et al.* 2022), it was selected as case study. One of the major risks identified with high likelihood of occurrence and associated with inhibition of OSM based on literature evidence was reduced angiogenesis. Extraction of sentences from our database containing a co-occurrence of OSM and angiogenesis was followed by classification of the sentences by our models. As demonstrated in Fig. 5 and Supplementary Table S3.2, major voting of strong relations indeed clearly indicates that (activation of) OSM increases angiogenesis, and that this effect is reduced upon inhibition.

## 4 Discussion

To facilitate target assessments for both therapeutic and toxicological outcomes, we developed an approach for multi-level classification of drug target–health effect relationships. As illustrated by the case studies, the classification models can be used both for target identification and for target safety assessments. For the former, the classification models allow evidence-based substantiation of hypotheses regarding the therapeutic value of a target. The mode of action associated with a beneficial health effect, captured by the target modulation class, indicates the type of intervention which should be pursued (e.g. inhibition or activation). For identifying safety liabilities arising from on-target effects, the target modulation class can be used to zoom in on the mode of action that has already been determined for the therapeutic intervention at hand.

In the design of our approach, we relied heavily on our experience in target assessments in which we want to (i) discard nonrelevant information (i.e. co-occurrences) as early as possible; (ii) distinguish indirect relationships from direct ones between health effects and target modulation; (iii) gain insight into the mode of action needed to either determine the therapeutic strategy or predict safety liabilities; (iv) weigh the evidence based on its certainty and on consensus between studies. This has resulted in the approach illustrated in Fig. 2. The results of the case studies show that, together, the models can correctly predict the effects of target modulation based on literature evidence. As such, the models can readily provide novel insights in the potential benefits and risks of novel drug targets.

To correctly capture target modulation, we have ensured that different therapeutic modalities have been incorporated in the training/validation set. Depending on their identity, they may induce target activation (e.g. agonist) or inhibition (e.g. antagonist). Modalities have been categorized as ‘altered’ if no specific direction is mentioned or associated with the term. This includes, e.g. antibodies (as opposed to blocking or agonistic antibodies) and modulators. The altered class also includes protein modifications such as cleavage. In the case of effects, ambiguous verbs were interpreted by the annotators to allow accurate cause–effect relation assessments. For example, improving health (increase) is different from improving fibrosis (decrease). As the named entities contain information for correct classification, masking was not used for classification levels 3A and B. Although in general the absence of masking may be expected to diminish the predictive power of models for unseen terms, the external validation set shows that this is not case for our models. In fact, absence of masking has a positive effect on the performance of the models for the two classification levels due to the need for interpretation of the terms annotated.

The BERT-based classification models can be applied in various ways. The case studies described in the current study illustrate the use of the classification models for single target assessments, either in the context of target efficacy or safety. These analyses are supported in the online TargetTri interface. However, off-line use allows analyses of the complete target space, e.g. in the context of a specific indication for target discovery. This was recently illustrated in a study on liver fibrosis (Venhorst *et al.* 2024). Here, we first identified all proteins in our ElasticSearch database that are directly linked to the pathogenesis of this disease. Subsequently, a disease network was created from proteins/genes showing the highest direct linkage with liver fibrosis. In a final step, the liver fibrosis disease network was explored to identify hub genes using clinical transcriptome data, which represent candidate drug target candidates. These hub genes were subsequently validated in vitro.

### 4.1 Limitations

The performance of the models for the training and validation set demonstrates that they are fit-for-purpose in classifying protein–health effect relations on multiple levels. One limitation of the current classification model is that sentences that are incorrectly classified on the first two levels are not taken into account in the performance of the other models. Another limitation of the model is that incorrect annotations of proteins and effects, often due to the use of abbreviations, are not considered. The acronym handling strategy (Supplementary Section S1) should largely mitigate such occurrences when using the TargetTri interface, as sentences with acronyms can be filtered out based on their confidence level. In addition, it is likely that the majority of such sentences merely represents a co-occurrence based on the grammatical structure.

We have captured a diversity of sentences in our training and test sets, on the one hand by random selection, and on the other by using filtering options to include e.g. various forms of target modulation and modalities. Nevertheless, the current dataset is unlikely to represent the complete variation in target modulation and in sentences describing protein–health effect relations in biomedical texts. Therefore, the implemented set of models will be further evaluated during our in-house target evaluations. Subject of more in-depth investigation will also be the model’s ability to correctly classify the direction of effects for ambiguous verbs that have not yet been seen by the model. Based on misclassified sentences, the models will be further fine-tuned in an iterative process. Further fine-tuning with additional data will also contribute to reducing the class imbalance. As such, the current models are not viewed as a static end result, but as a solid basis to further build upon. The applicability of the current models has been demonstrated in the case studies.

It should be noted that although the BERT-based classification models are fit-for-purpose, they only cover some of the aspects that are part of target evaluations. For example, the fact that the models are able to causally link potential targets, i.e. proteins/genes, to health effects through a specific mechanism of action does not necessarily mean these proteins are suitable as drug targets. This requires additional consideration, pertaining, e.g. to the druggability of the proteins, availability of tool compounds, feasible drug modalities, protein/gene expression levels and tissue distribution, and whether or not (high throughput) screening assays can be developed. These shortcomings of our approach can—at least in part—be overcome by applying additional relation classification models. For example, models that identify compound–target pairs can give insights in the druggability of a target. An advantage of combining distinct relation extraction models with overlapping entities is that this enables inference of novel relationships that may not be evident from the literature.

Another limitation of the models in their current form is that a domain expert still needs to interpret and combine the observed direct relationships into a strategic recommendation regarding the suitability of a target as drug target. With the emergence of generative AI, this may not be necessary in the near future. For example, the relation classification results could be incorporated in a RAG (Retrieval Augmented Generation) approach, in which generative language models could be deployed to derive meaningful interpretations from classified protein–health effect relations and their textual contexts. Additionally, such a RAG-based approach would also facilitate the integration of our classification results with other target-specific information present in our TargetTri database.

### 4.2 Related work

Several models for identifying relationships between biomedical entities such as drug-drug interactions (Liu *et al.* 2016) and drug–adverse effect relationships (Xu and Wang 2014) have been described. Aldahdooh *et al.* (2022) investigated the performance of BERT-based models for predicting the likelihood that a publication contains drug–target interactions to steer drug repurposing efforts (Aldahdooh *et al.* 2022). However, most relevant to our research is a study on a single, fine-tuned PubMedBERT model described by Lee *et al.* (2022) for classifying relations between twelve categories of biomedical entities into eight classes. Several of these biomedical categories overlap fully or partially with our drug target and health effect taxonomies. With a per class F1 score of 0.840 ± 0.014, the model shows similar but somewhat lower performance as those reported here. However, our study aim, and therefore the used approach, differs. The present study is aimed at classifying all relations deemed relevant for drug target identification and assessment, while Lee’s work (Lee *et al.* 2022) focuses on classifying only causal relations between biomedical entities (Supplementary Section S4). As such, differences between both approaches include the ability to distinguish contextual information from co-occurrences, the individual classification of target modulation and the direction of the effect, the specific classification of noncausal (associative) relations, and the classification of the certainty of the relationship. In our experience, contextual information (i.e. weak relations) can contain valuable information for drug target profiling. For example, certain effects may be specific to, or exacerbated by, the presence of a disease, or occur only in a specific disease model. Strong relations, such as disease associations, which are not considered causal in Lee’s model but are explicitly classified in our model, are also an integral part of target discovery and evaluation activities. Our models furthermore allow weighing of evidence in two ways: by means of the certainty class, and by examining the consensus of reported relations in different studies.

## 5 Conclusions

The BioBERT-based models developed in the current study show robust performance in classifying drug target–health effect relationships on a highly detailed level. Collectively, these models can significantly improve target identification and assessment activities and provide quick insights in how target modulation is affecting health and disease, as illustrated for ADAM33 and OSM. The fine-tuned models have been made available via the TargetTri platform, which allows on-the-fly queries of target-mediated health effects reported in PubMed. With the current models and underlying classification levels, automated data interpretation comes within reach and is envisaged as one of the next steps in optimizing our drug target assessment pipeline for drug discovery. Integration of additional relation extraction models covering the distinct entity pairs relevant for drug development will also be pursued to allow inference of novel insights for target discovery and evaluation.

## Supplementary Material

vbaf043_Supplementary_Data

## Data Availability

The implemented classification models are available via the TargetTri platform (www.targettri.com), which is free to use for academics, The training and validation datasets generated in this study are available at https://diamonds.tno.nl/ttdata and can be downloaded after logging into the diamonds platform.
