# Supplementary material for: Drug target assessments: classifying target modulation and associated health effects using multi-level BERT-based classification models
Source: Bioinform Adv. 2025 Mar 8;5(1):vbaf043. doi: 10.1093/bioadv/vbaf043 (PMC11919816; doi:10.1093/bioadv/vbaf043)
Supplement: vbaf043_Supplementary_Data [file vbaf043_supplementary_data.pdf]

*Supplementary material*

# Drug target assessments: Classifying target modulation and associated health effects using multi-level BERT-based classification models

Jennifer Venhorst[1*#] and Gino Kalkman[1*]

[1]Biomedical and Digital Health, The Netherlands Organization for Applied Scientific Research (TNO), Princetonlaan 9, 3584 CB Utrecht, The Netherlands

*Authors contributed equally to this paper

# corresponding author; e-mail: Jennifer.mccormack@tno.nl

# Supplementary Material

## 1 Data processing pipeline

### Taxonomies

Hierarchical taxonomies were created for the categories proteins/genes (size = ~200.000), organ systems/cells (size = ~7000), and health effects (size = ~80.000), with the latter covering disease, pathology, physiology, (bio)markers of disease and cellular processes. The UniProt database (Bateman et al., 2023) was used as the primary basis to structure the gene/protein taxonomy, whereas the MeSH (Lipscomb, 2000) tree was used as the primary basis for the other categories. Protein/gene names were expanded with data from other sources, including HGNC (Seal et al., 2023), NCBI (Sayers et al., 2022), and ChEMBL (Zdrazil et al., 2023). The CTD database (Davis et al., 2023) was used to expand the health effect taxonomy. Abbreviations of terms were manually annotated as such in each respective taxonomy. Cross-mapping, as provided by the various databases, was used to hierarchically position new terms within the taxonomy. In the absence of mappings, manual assignments were performed by experts. Further enrichment of taxonomies was achieved in two ways: 1) manual curation of terms based on expert knowledge and specific domain expertise performed by at least three annotators; 2) automated enrichment with approaches based on word embeddings (e.g. Word2Vec (Mikolov et al., 2013)) and Large Language Models (e.g. GPT), again followed by manual curation.

### Acronym handling

Use of acronyms is a well-known feature of biomedical texts which complicates the task of entity recognition. Many acronyms may refer not only to multiple biological entities, but also to entities outside the biological domain (e.g.: *HF* may stand for the biological effect *heart failure* as well as for the technical term *high frequency*). As a result, simply retrieving data based on the occurrence of such an acronym usually results in a lot of noise, making it cumbersome to filter out the relevant literature.

For these reasons, we implemented the acronym-detection algorithm introduced by Saneesh and Nazeer (Saneesh & Nazeer, 2013), who have based their views to a great extent on the work of Schwartz and Hearst (Schwartz & Hearst, 2003). This algorithm helps identify the acronym-definition pairs in a text based on parameters such as the length of acronym and definition candidate words and the presence of parentheses and specific alphanumeric characters.

### Annotation process

The above-mentioned taxonomies as well as the acronym resolution algorithms were used to identify and mark all possible references to drug targets and health effects in our corpus of PubMed titles and

abstracts. Each sentence in this corpus was automatically annotated. A set of knowledge-driven rules ensured that annotations did not conflict or overlap by preventing, for instance, embedding of one entity in another one (e.g.: since *tumor necrosis factor* is a protein name, *necrosis* will not be marked as health effect in this context). Assisted by the above-mentioned algorithms for identifying acronym-definition pairs, acronyms were categorized based on the level of confidence that they did or did not refer to an entity defined in our taxonomies. This categorization has been implemented as a filter for the results set viewed by the user in the TargetTri platform.

## PubMed data pre-processing

Our database contains annotated texts from all PubMed abstracts published since the year 2000, which is a text corpus of more than 24 million abstracts. Daily downloads are being made from the PubMed FTP server to stay up-to-date (*PubMed Index of Daily Update Files*, 2024). In order to be able to identify and classify the relations between drug targets and health effects in these abstracts, some basic pre-processing steps were required. Thus, we used libraries from the Natural Language Toolkit for tokenization of sentences and words, part-of-speech tagging and lemmatization (Bird et al., 2016). A schematical representation of our PubMed data processing pipeline is depicted in Figure 1.

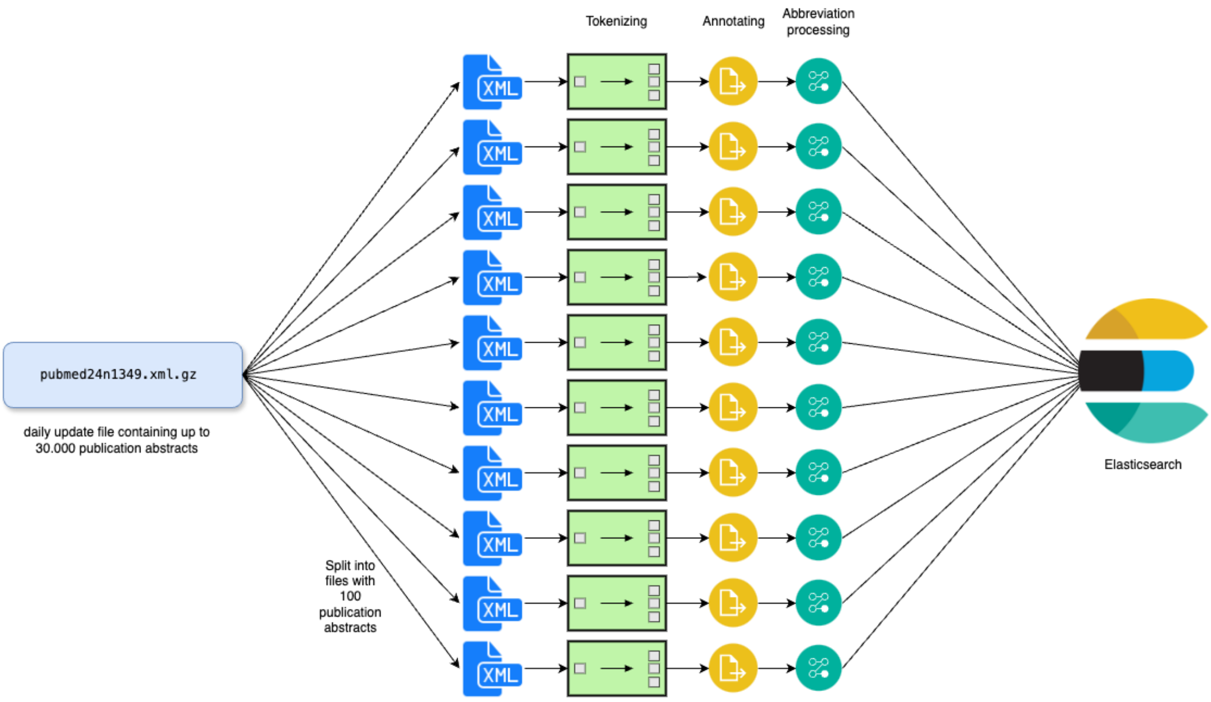


Figure 1.1 Schematic presentation of the TargetTri pipeline for processing PubMed data. Every day, zip files containing up to 30.000 publication abstracts are downloaded from the PubMed FTP server. Subsequently, the abstracts are processed in batches of 100 abstracts on a High Performance Cluster. Abstracts are tokenized into sentences and sentences are tokenized into words. After the word tokens have been lemmatized, TargetTri ontologies are used to automatically annotate protein references,

health effects and organ systems in the abstracts. Acronyms and their definitions are identified with dedicated acronym detection algorithms. Finally, both the original data and the annotations are stored in ElasticSearch to allow for efficient use of the data in the TargetTri platform.

## Classification scheme

The classification scheme that was applied by the annotators encompasses three levels (Manuscript Figure 1) and is described and exemplified below. In the examples, drug targets (i.e. proteins/genes including receptors, enzymes, hormones etc.) are shown in **bold**, and health effects are underlined.

### *Classification level 1: co-occurrence vs relation*

The first classification level determines whether the annotated drug target and health effect entities constitute a co-occurrence or a relation. In case of a co-occurrence, no additional classifications are performed.

Table 1.1 Classification level 1: presence of relation.

| Class | Explanation | Example |
|---|---|---|
| No | Simple co-occurrence: the sentence does not describe a relation between drug target and health effect | *This association persisted in statistical models adjusted for age, body-mass index, insulin sensitivity , and* ***C-reactive protein****.* |
| Yes | The sentence describes an actual relation between drug target and health effect | *Type A insulin resistance syndrome is caused by mutations in the* ***insulin receptor (INSR)*** *gene.* |

### *Classification level 2: strength of the relation*

This classification level allows the distinction between causal and contextual relationships. In case of a contextual relationship (class: weak), no further classification is performed. Strong relations are further classified based on the direction of target and effect modulation and the certainty of the described relation (levels 3A, 3B and 3C).

Table 1.2 Classification level 2: strength of relation.

| Class | Explanation | Example |
|---|---|---|
| Strong | The sentence refers to a clear and direct link between drug target and health effect | *The regulatory (…)* ***cyclin-dependent kinases*** *(…) control the cell cycle progression.* |
| Weak | The sentence describes a contextual link between drug target and health effect or the intention to investigate the existence of a relation between drug target and health effect | ***β-catenin*** *was also downregulated by miR-195 in colon cancers.*<br><br>*Here, we investigated the effect of* ***UCP*** *gene transfer on therapeutic angiogenesis.* |

### *Classification level 3A: Modulation of the target*

The direction of target modulation is captured by this classification level. Modulation can either be on the level of the gene (e.g. up- or downregulation) or the protein (e.g. activation or inhibition). When no specific modulation is mentioned in the sentence, the class is set to 'Neutral'.

Table 1.3 Classification level 3A: modulation of the target. Signal words indicating modulation are highlighted in grey.

| Class | Explanation | Example |
|---|---|---|
| Positive | Activation of the drug target is associated with the health effect | *Stimulation of* ***Tumor Necrosis Factor Alfa (TNF-α)*** *(…) induced zebrafish immunotoxicity.* |
| Negative | Inhibition of the drug target is associated with the health effect | *We noticed that knockdown of* ***ZNF460*** *(…) increased apoptosis rate of AML cells.* |
| Altered | Regulation of the drug target (other than activation or inhibition) is associated with the health effect | *We report that targeting the Notch ligand* ***Jagged1*** *by a monoclonal antibody reduces (…) and vascular leakage in an experimental CNV mouse model.* |
| Variant | Genetic modification of the target is associated with the health effect | *Progressive familial intrahepatic cholestasis type 2 (PFIC2) is an ultra-rare* |

| | | |
|---|---|---|
| | | *disease caused by mutations in the **ABCB11** gene.* |
| Neutral | No specific modulation of the target is mentioned in the sentence | *These data highlight the ability of **irisin** to alleviate CIRI in vivo and in vitro.* |

### *Classification level 3B: Direction of the effect*

Essential to drug target assessment is the direction of the health effect associated with its modulation, which is represented by this classification level. A health effect can constitute several types, e.g. a biological effect, disease, or a (bio)marker level.

Table 1.4 Classification level 3B: direction of health effect. Signal words indicating the direction of the health effect upon target modulation are highlighted in grey.

| **Class** | **Explanation** | **Example** |
|---|---|---|
| Decrease | (Modification of) the drug target causes a decrease of the health effect | *Elevation of **p27Kip1** protein level is found to be the sole requirement for the inhibition of cellular proliferation (…).* |
| Increase | (Modification of) the drug target results in an increase of the health effect | ***Parkin** increases the risk of colitis.* |
| Neutral | (Modification of) the drug target results in an unspecified change of the health effect | *Controlling cell proliferation by targeting **cyclin-dependent kinase 6** (…).* |

### *Classification level 3C: Certainty of the relation*

Authors often express a certainty for the observations made. Signal words such as “may” and “potential” have been interpreted as indicators of an uncertain observation. The absence of an investigated relationship is captured by the ‘negative’ class.

Table 1.5 Classification level 3C: certainty of the relation.

| **Class** | **Explanation** | **Example** |
|---|---|---|
| Positive | The sentence states that there is a clear relation between the target and the effect | ***Adiponectin** reverses β-Cell damage and impaired insulin secretion induced by obesity.* |

| | | |
|---|---|---|
| Negative | The sentence states that the investigated drug target – effect relation is not observed | *Our data underline that **β-synuclein** is not a direct marker of Aβ pathology.* |
| Uncertain | The sentence states that there may potentially be a relation between the target and the effect | *In particular, the inhibition of the α9-containing **nAChRs** by α-conotoxins may be a pathway to alleviate neuropathic pain.* |

## Data set statistics

Table 1.6 Distribution of classes in the training set.

| **Classification level** | **Class** | **Count** |
|---|---|---|
| Presence of relation | Yes | 2545 |
| | No | 932 |
| Strength of relation | Strong | 2006 |
| | Weak | 539 |
| Modulation of target | Positive | 350 |
| | Negative | 596 |
| | Altered | 250 |
| | Variant | 162 |
| | Neutral | 648 |
| Direction of effect | Increase | 576 |
| | Decrease | 610 |
| | Neutral | 820 |
| Certainty of relation | Positive finding | 1632 |
| | Negative finding | 133 |
| | Uncertain finding | 241 |

Table 1.7 Distribution of classes in the external validation set.

| **Classification level** | **Class** | **Count** |
|---|---|---|
| Presence of relation | Yes | 322 |
| | No | 92 |
| Strength of relation | Strong | 247 |
| | Weak | 75 |

| | | |
|---|---|---|
| Modulation of target | Positive | 59 |
| | Negative | 63 |
| | Altered | 27 |
| | Variant | 33 |
| | Neutral | 65 |
| Direction of effect | Increase | 79 |
| | Decrease | 56 |
| | Neutral | 112 |
| Certainty of relation | Positive finding | 168 |
| | Negative finding | 53 |
| | Uncertain finding | 26 |

# 2 Model parameters and performance

## Model Parameters

Table 2.1 Hyperparameters used in the final BioBERT classification models.

| **Hyperparameter** | **Value** |
|---|---|
| Learning rate | 2e-5 |
| Per-device training batch size | 16 |
| Per-device evaluation batch size | 16 |
| Number of training epochs | 5 |
| Weight decay | 0.01 |

## Performance metrics

Table 2.2 Confusion matrix (top) and derived performance metrics (bottom) used in this study.

| | | **Predicted class** | |
|---|---|---|---|
| | | *Positive* | *Negative* |
| **Actual class** | *Positive* | True Positive (TP) | False Negative (FN) |
| | *Negative* | False Positive (FP) | True Negative (TN) |

| Metric | Formula | Explanation |
|---|---|---|
| **Accuracy** | *(TP+TN) / (TP + TN + FP + FN)* | Proportion of correctly predicted classes |
| **Precision** | *TP / (TP + FP)* | Ratio of true positive predictions to the total number of positive predictions |
| **Recall** | *TP / (TP + FN)* | Ratio of true positive predictions to the total number of actual positive instances |
| **F1** | *(2 * Precision * Recall) / (Precision + Recall)* | Harmonic mean of precision and recall |

## Performance training set per class

Table 2.3 Performance of the model predicting the presence of a relation across five folds for the training set.

| Class | Precision | Recall | F1 |
|---|---|---|---|
| Yes | 0.95 ± 0.010 | 0.98 ± 0.006 | 0.96 ± 0.007 |
| No | 0.95 ± 0.017 | 0.85 ± 0.029 | 0.89 ± 0.021 |

Table 2.4 Performance of the model predicting the strength of the relation across five folds for the training set.

| Class | Precision | Recall | F1 |
|---|---|---|---|
| Strong | 0.97 ± 0.006 | 0.98 ± 0.005 | 0.97 ± 0.004 |
| Weak | 0.92 ± 0.018 | 0.88 ± 0.025 | 0.90 ± 0.017 |

Table 2.5 Performance of the model predicting the direction of target modulation across five folds for the training set.

| Class | Precision | Recall | F1 |
|---|---|---|---|
| Positive | 0.9 ± 0.027 | 0.91 ± 0.031 | 0.91 ± 0.020 |
| Negative | 0.94 ± 0.023 | 0.96 ± 0.014 | 0.95 ± 0.017 |
| Altered | 0.84 ± 0.048 | 0.82 ± 0.041 | 0.83 ± 0.029 |
| Variant | 0.91 ± 0.071 | 0.89 ± 0.065 | 0.9 ± 0.047 |
| Neutral | 0.94 ± 0.019 | 0.92 ± 0.019 | 0.93 ± 0.014 |

Table 2.6 Performance of the model predicting the direction of the effect across five folds for the training set.

| Class | Precision | Recall | F1 |
| --- | --- | --- | --- |
| Increase | 0.88 ± 0.016 | 0.87 ± 0.008 | 0.87 ± 0.007 |
| Decrease | 0.91 ± 0.019 | 0.94 ± 0.017 | 0.92 ± 0.010 |
| Neutral | 0.92 ± 0.019 | 0.91 ± 0.005 | 0.92 ± 0.008 |

Table 2.7 Performance of the model prediction the relation certainty across five folds for the training set.

| Class | Precision | Recall | F1 |
| --- | --- | --- | --- |
| Positive finding | 0.98 ± 0.006 | 0.98 ± 0.010 | 0.98 ± 0.004 |
| Negative finding | 0.92 ± 0.038 | 0.95 ± 0.039 | 0.93 ± 0.012 |
| Neutral | 0.88 ± 0.053 | 0.9 ± 0.039 | 0.89 ± 0.033 |

## Performance external validation set per class

Table 2.8 Performance of the model predicting the presence of a relation for the external validation set.

| Class | Precision | Recall | F1 |
| --- | --- | --- | --- |
| Yes | 0.96 | 0.97 | 0.97 |
| No | 0.89 | 0.87 | 0.88 |

Table 2.9 Performance of the model predicting the strength of the relation for the external validation set.

| Class | Precision | Recall | F1 |
| --- | --- | --- | --- |
| Strong | 0.92 | 0.95 | 0.94 |
| Weak | 0.81 | 0.75 | 0.78 |

Table 2.10 Performance of the model predicting the direction of target modulation for the external validation set.

| Class | Precision | Recall | F1 |
|---|---|---|---|
| Positive | 0.89 | 0.95 | 0.92 |
| Negative | 0.94 | 0.92 | 0.93 |
| Altered | 0.88 | 0.81 | 0.85 |
| Variant | 0.78 | 0.85 | 0.81 |
| Neutral | 0.9 | 0.85 | 0.87 |

Table 2.11 Performance of the model predicting the direction of the effect for the external validation set.

| Class | Precision | Recall | F1 |
|---|---|---|---|
| Increase | 0.83 | 0.85 | 0.84 |
| Decrease | 0.85 | 0.91 | 0.88 |
| Neutral | 0.93 | 0.88 | 0.91 |

Table 2.12 Performance of the model prediction the relation certainty for the external validation set.

| Class | Precision | Recall | F1 |
|---|---|---|---|
| Positive finding | 0.98 | 0.99 | 0.99 |
| Negative finding | 1.0 | 0.96 | 0.98 |
| Uncertain finding | 0.89 | 0.92 | 0.91 |

## Macro-average performance external validation set for 3 types of BERT-based models

Table 2.13 Comparison of macro-average performance of Generic BERT, PubMedBERT and BioBERT for the external validation set.

| Classification level | Model | Accuracy | Precision | Recall | F1 |
|---|---|---|---|---|---|
| 1 | Generic BERT | 0.84 | 0.77 | 0,78 | 0.77 |
| | PubMedBERT | 0.94 | 0.92 | 0.89 | 0.91 |
| | **BioBERT** | 0.95 | 0.93 | 0.92 | **0.92** |
| 2 | Generic BERT | 0.86 | 0.84 | 0.85 | 0.84 |

| | | | | | |
|---|---|---|---|---|---|
| | PubMedBERT | 0.89 | 0.85 | 0.86 | 0.85 |
| | **BioBERT** | 0.90 | 0.87 | 0.85 | **0.86** |
| 3A | Generic BERT | 0.81 | 0.79 | 0.78 | 0.78 |
| | **PubMedBERT** | 0.89 | 0.88 | 0.88 | **0.88** |
| | **BioBERT** | 0.89 | 0.88 | 0.88 | **0.88** |
| 3B | Generic BERT | 0.89 | 0.88 | 0.88 | 0.88 |
| | **PubMedBERT** | 0.91 | 0.9 | 0.91 | **0.91** |
| | BioBERT | 0.88 | 0.87 | 0.88 | 0.88 |
| 3C | Generic BERT | 0.96 | 0.93 | 0.93 | 0.93 |
| | PubMedBERT | 0.96 | 0.93 | 0.92 | 0.92 |
| | **BioBERT** | 0.98 | 0.96 | 0.96 | **0.96** |

# 3 Case studies

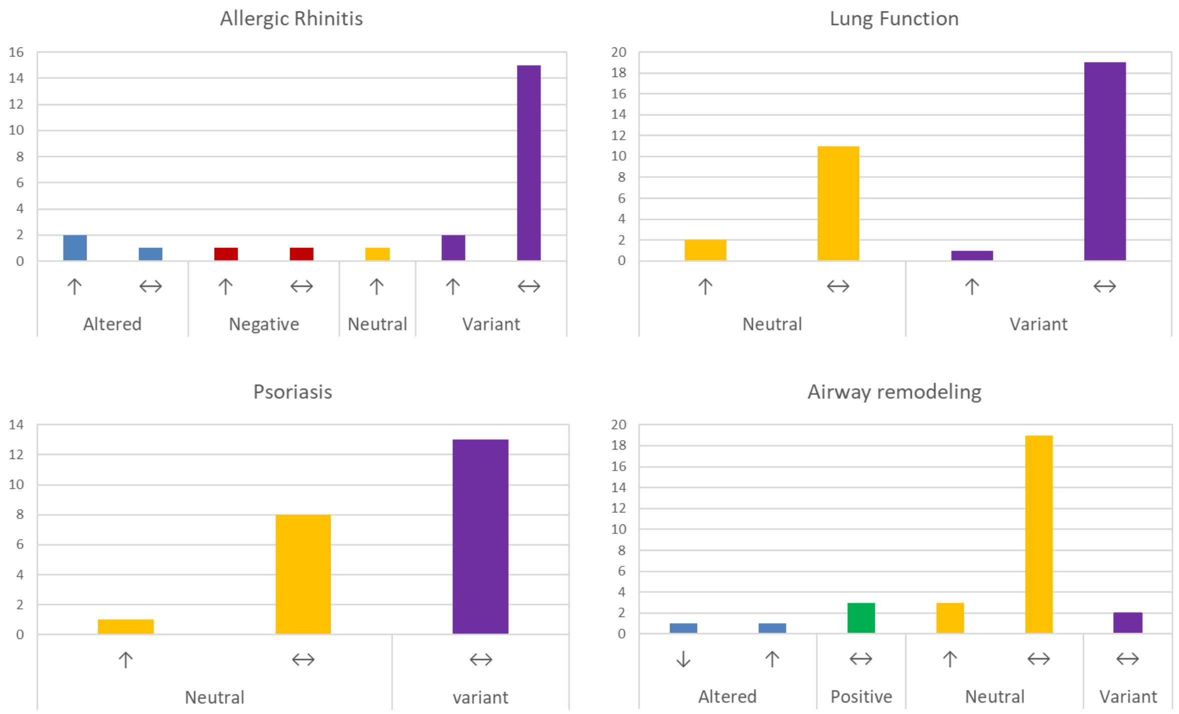


Figure 3 Classification of top-ranked health effects with ADAM33 for strong relations (class level 2: strong). Target modulation (classification level 3A) is indicated by color and text; the direction of the effect (classification level 3B) is indicated by arrows. The y-axis shows the number of classified sentences.

Table 3.1 Classification of strong relations (class level 2: strong) of ADAM33 with psoriasis and breast cancer.

| PMID | Sentence | Class 3A | Class 3B | Class 3C |
|---|---|---|---|---|
| **Psoriasis** | | | | |
| 18560587 | Interestingly , the 2 ADAM33 SNPs associated with psoriasis in the present analysis were part of the 3-SNPs haplotypes showing the strongest associations in the initial study . | variant | neutral | positive finding |
| 18560587 | The identification of a pleiotropic effect of ADAM33 on asthma and psoriasis may contribute to the understanding of these common immune-mediated diseases . | neutral | neutral | uncertain finding |
| 22269827 | ADAM33 polymorphisms are associated with psoriasis in the northeastern Chinese Han population . | variant | neutral | positive finding |

| | | | | |
|---|---|---|---|---|
| 24562625 | Association of ADAM33 gene polymorphisms with psoriasis in a northeastern Chinese population . | variant | neutral | positive finding |
| 30160137 | ADAM33 V4 C/G rs2787094 polymorphism was not associated with psoriasis risk in the Turkish population . | variant | neutral | negative finding |
| 17878941 | ADAM33 , a new candidate for psoriasis susceptibility . | neutral | neutral | positive finding |
| 24562625 | Recently , ADAM33 has been suspected to be associated with PS . | neutral | neutral | uncertain finding |
| 17878941 | The identification of ADAM33 as a psoriasis susceptibility gene identified by positional cloning in an outbred population should provide insights into the pathogenesis and natural history of this common disease . | neutral | neutral | positive finding |
| 20491578 | Association of ADAM33 polymorphisms and susceptibility to psoriasis . | variant | neutral | positive finding |
| 20491578 | Recently , it has been reported that ADAM33 contributed to PS risk in the French population and white North Americans . | neutral | increase | positive finding |
| 20491578 | Our data suggest that the ADAM33 gene may be associated with PS risk in the Chinese population . | variant | neutral | uncertain finding |
| 18560587 | This suggests independent effects of ADAM33 and PSORS1 on psoriasis . | neutral | neutral | negative finding |
| 18560587 | This is the first study that replicates an association between genetic variants in ADAM33 and psoriasis . | variant | neutral | positive finding |
| 22269827 | ADAM33 as a psoriasis susceptibility gene in the Han population of northeastern China . | variant | neutral | positive finding |
| 22269827 | A disintegrin and metalloprotease 33 ( ADAM33 ) has recently been suspected to be associated with psoriasis . | neutral | neutral | uncertain finding |
| 30160137 | There was no significant difference in ADAM33 genotype and allele distributions between psoriasis and control groups ( p > 0.05 ) . | variant | neutral | negative finding |
| 24562625 | Our data suggest that the ADAM33 polymorphisms may be associated with PS in the northeastern Chinese population . | variant | neutral | uncertain finding |
| 18560587 | Replication of association between ADAM33 polymorphisms and psoriasis . | variant | neutral | positive finding |
| 18560587 | Polymorphisms in ADAM33 , the first gene identified in asthma by positional cloning , have been recently associated with psoriasis . | variant | neutral | positive finding |
| 18560587 | The rs512625 SNP in ADAM33 was found associated with psoriasis at p = 0.01 , the usual threshold required for replication ( OR [ 95 % CI ] for heterozygotes compared to the reference group of homozygotes for the most frequent allele = 0.61 [ 0.42 ; 0.89 ] ) . | variant | neutral | positive finding |

| | | | | |
|---|---|---|---|---|
| 17878941 | Thus , the gene ADAM33 was found to be significantly associated with psoriasis in this family set ( The best association was on a 3-SNP haplotype P = 0.00004 , based on 1,000,000 permutations ) . | neutral | neutral | positive finding |
| **Breast cancer / breast tumor** | | | | |
| 28294120 | Down regulation of ADAM33 as a Predictive Biomarker of Aggressive Breast Cancer . | negative | neutral | positive finding |
| 28294120 | We used 212 breast tumor samples and lower levels of ADAM33 were correlated with TNBC and basal-like markers . | negative | neutral | positive finding |
| 30226539 | Breast cancer is associated with methylation and expression of the a disintegrin and metalloproteinase domain 33 ( ADAM33 ) gene affected by endocrine-disrupting chemicals . | altered | neutral | positive finding |

Table 3.2 Classification of strong relations (class level 2: strong) of OSM with angiogenesis. Undetermined = neutral classification.

| PMID | Sentence | Class 3A | Class 3B | Class 3C |
|---|---|---|---|---|
| 38511182 | Oncostatin M mediates cardioprotection via angiogenesis in ischemic heart disease . | neutral | increase | positive finding |
| 38586430 | Corrigendum to `` Oncostatin M mediates cardioprotection via angiogenesis in ischemic heart disease '' | neutral | increase | positive finding |
| 21174212 | Our findings that OSM , which is present in human atherosclerotic lesions and correlates with VEGF expression , stimulates production of VEGF by human coronary artery and aortic SMC indicate that OSM could contribute to plaque angiogenesis and destabilization . | neutral | increase | uncertain finding |
| 35064579 | Cell culture experiments indicated that OSM upregulation in hepatic cancer cells contributes to HCC progression by inducing epithelial-to-mesenchymal transition and increased invasiveness of cancer cells as well as by inducing angiogenesis , which is of critical relevance . | positive | increase | positive finding |
| 26146616 | OSM Enhances Angiogenesis and Improves Cardiac Function after Myocardial Infarction . | neutral | increase | positive finding |
| 26146616 | In conclusion , OSM treatment preserved cardiac function , inhibited apoptosis and fibrosis , and stimulated angiogenesis via upregulating VEGF and bFGF in infarct border zone of ischemic myocardium , indicating that | positive | increase | positive finding |

| | | | | |
|---|---|---|---|---|
| | OSM could be a novel therapeutic target for MI . | | | |
| 17009243 | Oncostatin M induces angiogenesis and cartilage degradation in rheumatoid arthritis synovial tissue and human cartilage cocultures . | neutral | increase | positive finding |
| 17525365 | We speculate that the inflammatory cytokines IL-6 and OSM might support angiogenesis during adipose tissue growth by upregulating VEGF . | neutral | increase | uncertain finding |
| 25252914 | Conversely , selective inhibition of OSM by neutralizing antibody and Jak family kinases by tofacitinib inhibited STAT3 signaling , peritumoral angiogenesis , and cellular scattering . | negative | decrease | positive finding |
| 26146616 | Oncostatin M ( OSM ) has been reported to stimulate angiogenesis by upregulating VEGF and bFGF , implying that it could be a therapeutic strategy in treating ischemic diseases . | neutral | increase | positive finding |
| 35064579 | Oncostatin M is overexpressed in NASH-related hepatocellular carcinoma and promotes cancer cell invasiveness and angiogenesis . | positive | increase | positive finding |
| 20650266 | OSM , a cytokine of the IL-6-type cytokine family , regulates inflammatory processes ( like the acute phase response ) , tissue remodeling , angiogenesis , cell differentiation and proliferation . | neutral | neutral | positive finding |
| 38511182 | Importantly , HF patients with IHD had higher OSM levels , and those with collateral flow showed the even higher levels , indicating a potential involvement in angiogenesis . | positive | neutral | uncertain finding |
| 10967551 | Together , these and other recent studies support a role for OSM in modulating the different phases of angiogenesis . | neutral | neutral | positive finding |
| 35997155 | After the gelation in situ , the OSM-loaded hydrogel exhibited continuous and localized release of OSM in response to specific pH and changes in MI rats , thereby accelerating angiogenesis and proliferation of cardiomyocytes , inhibiting myocardial fibrosis and improving cardiac function effectively . | positive | increase | positive finding |
| 23068100 | Previously we have shown that expression of the inflammatory cytokine , Oncostatin-M ( OSM ) , in human atherosclerotic plaques correlated with increased microvessel density , indicating a role for OSM in promoting plaque angiogenesis . | neutral | increase | positive finding |
| 16204061 | Whereas oncostatin M can inhibit the proliferation of breast cancer cells in vitro , recent studies suggest that oncostatin M may promote tumor progression by enhancing angiogenesis and metastasis . | neutral | increase | uncertain finding |
| 20088942 | Our data , showing the effects of OSM on the Ang-Tie system in endothelial | neutral | neutral | positive finding |

| | | | | |
|---|---|---|---|---|
| | cells , in hearts of mice , and in human heart tissue , provide yet another link between inflammation and angiogenesis . | | | |
| 11460521 | Here we show that OSM acts ( 1 ) by inducing the secretion involved in invasion of the vessel wall by monocytes ; ( 2 ) by inducing angiogenesis it promotes plaque destabilization , rupture , and consequently thrombosis ; and ( 3 ) by decreasing fibrinolysis on macrovascular endothelial cells . | neutral | increase | positive finding |
| 16023359 | We find that OsM strongly and specifically affects the expression of many genes , in particular those involved with innate immunity , angiogenesis , adhesion , motility , tissue remodeling , cell cycle and transcription . | neutral | neutral | positive finding |
| 25954856 | Oncostatin M activates STAT3 to promote endometrial cancer invasion and angiogenesis . | neutral | increase | positive finding |
| 36458801 | Surprisingly some STAT3 activators including the IL6 cytokine member oncostatin M ( OSM ) enhance angiogenesis whereas others like ciliary neurotropic factor ( CNTF ) reduce it . | neutral | increase | positive finding |
| 34702860 | Macrophages can acquire different functional phenotypes and promote mesenchymal stem cell ( MSC ) osteogenic differentiation , chondrogenic differentiation , and angiogenesis by expressing cytokines and other factors such as the transforming growth factor-β1 ( TGF-β1 ) , bone morphogenetic protein ( BMP ) , activin A ( Act A ) , oncostatin M ( OSM ) , substance P ( SP ) , neurotrophin-3 ( NT-3 ) , and vascular endothelial growth factor ( VEGF ) . | positive | increase | positive finding |
| 17009243 | These data suggest that OSM promotes angiogenesis and endothelial cell migration and potentiates the effects of IL-1beta in promoting extracellular matrix turnover and human cartilage degradation . | neutral | increase | positive finding |

## 4 Comparison related work

The tables below illustrate how the top classification levels and classes in our study relate to those defined in the model by Lee (Lee et al., 2022). In the latter study, relations between biomedical entities were classified in eight classes based on their causal nature and direction (Table 4.1). Lee's model primarily focusses on the prediction of whether or not the identified relation is causal ('directed link') or not ('undirected link'; i.e. a correlation without causation revealed), with further classification if the

sentence allows quantification of entity modulation. Because of this focus on causal relations, multiple classes and levels assigned in our approach fall within the ‘undirected link’ class in the model by Lee: a co-occurrence (classification level 1), weak relations (classification level 2) and strong relations with an unspecified or ‘neutral’ direction of the effect (classification level 3B) (see additional file 3). The target modulation classes ‘altered’ and ‘variant’ and relations with explicit target modulation in combination with an ‘neutral’ effect are not specifically classified. For the latter, the absence of this specification is partly covered by the addition of other biomedical entities. For example, in our model the sentence ‘**Toll-like receptor** (TLR) agonists induce metabolic reprogramming’ is classified as a positive (level 3A) and increase (level 3B) class. In the model by Lee, ‘Toll-like receptor (TLR) agonists’ are recognized as a compound entity and have a positive causal relationship with metabolic programming. The certainty of relation is not predicted by the model described by Lee. It should furthermore be noted that in our model indirect relationships are viewed as strong relations. Thus, a sentence such as ‘Early studies revealed that cigarette smoke promotes gastric cancer growth through the induction of cyclooxygenase-2 **COX-2**’ is classified into the positive (level 3A) increase (level 3B) class. In Lee’s study, this sentence is assigned to the ‘undirected link’ class.

Table 4.1 Eight classes defined in the model by Lee between two biomedical entities.

| | | |
|---|---|---|
| Undirected link | = | = |
| Directed link | Positive Cause | Positive increase |
| | | Negative decrease |
| | Negative Cause | Positive decrease |
| | | Negative increase |

Table 4.2 Comparison of several classification levels and classes applied in our approach with those described by Lee (Lee et al., 2022) using examples provided in the corresponding paper and supplementary material. Targets are indicated in bold; effects are underlined.

| **Level** | **Class** | **Corresponding class Lee** | **Example (*class by Lee*)** |
|---|---|---|---|
| 1 | No | Undirected link | A549 cells treated with AE-AS caused activation of **caspase-9** and -3, and AE-AS-induced apoptosis could be inhibited by the broad-spectrum caspase inhibitor, z-VAD-fmk. *(undirected link)* |

| | | | |
|---|---|---|---|
| | Yes | Any Class | All the cognitive tests completed in 1983 predicted onset of dementia and <u>Alzheimer's disease</u> up to 11 years later, as did **APOE** genotype. *(undirected link)* |
| 2 | Weak | Undirected link | Increased expression of **TLR9** associated with pro-inflammatory S100A8 and IL-8 in <u>diabetic wounds</u> could lead to unresolved inflammation in type 2 diabetes mellitus T2DM cases with impaired wound healing. *(undirected link)* |
| | Strong | Any class | These studies have demonstrated that **PEPCK1** is integral to coordinating <u>cellular metabolism</u> in the liver and adipose tissue, although it does not directly affect hepatic glucose production or adipose glyceroneogenesis. (*directed link*) |
| 3A/3B | Neutral/neutral | Directed or undirected link | These studies have demonstrated that **PEPCK1** is integral to coordinating <u>cellular metabolism</u> in the liver and adipose tissue, although it does not directly affect hepatic glucose production or adipose glyceroneogenesis. (*directed link*)<br><br>Endocan, **apelin** and endoglin are thought to be associated with endothelial dysfunction, <u>angiogenesis</u> and inflammation. (*undirected link*) |
| | Positive/neutral | Directed or undirected link | Because overproduction of **p52** is associated with <u>lymphoid hyperplasia</u> and transformation, deregulation of p100 processing may be part of the oncogenic mechanism of HTLV-I. *(undirected link)* |
| | Negative/neutral | Directed or undirected link | Decreases in the expression of **CGRP** and galanin mRNA in |

| | | | |
|---|---|---|---|
| | | | central and peripheral neurons related to the control of blood pressure following experimental hypertension in rats. (*undirected link*) |
| | Altered/neutral | Directed or undirected link | CpG island hypermethylation of the DNA repair enzyme **methyltransferase** predicts response to temozolomide in primary gliomas. (*undirected link*) |
| | Variant/neutral | Directed or undirected link | All the cognitive tests completed in 1983 predicted onset of dementia and Alzheimer's disease up to 11 years later, as did **APOE** genotype. (*undirected link*) |
| | Variant/increase | Positive cause | In addition, **TUBB1** mutations caused the formation of macroplatelets and hyperaggregation of human platelets after stimulation by low doses of agonists. (*positive cause*) |
| | Neutral/increase | Positive cause | Moreover, our results suggest that androgens and the growth factors Insl3 and **Igf3** stimulate spermatogenesis via independent pathways. (positive cause) |
| | Neutral/decrease | Negative cause | In contrast, **PEPCK1** ASO decreased the white adipose tissue mass in HFF rats but without altering basal rates of lipolysis, de novo lipogenesis, or glyceroneogenesis in vivo. (*negative cause*) |
| | Positive/increase | Positive increase | Functionally, over-expression of NR4A3 attenuated proliferation of cancer cells and promoted apoptosis by augmenting the expression of pro-apoptotic genes, **PUMA** and Bax. (*positive increase*) |
| | Negative/increase | Negative increase | **DCLK1-B** depletion impairs cancer stemness resulting in reduced survival potential and increased **apoptosis**, thus sensitizing colorectal cancer to chemoradiation. *(negative increase)* |

## 5 References


Bateman, A., Martin, M. J., Orchard, S., Magrane, M., Ahmad, S., Alpi, E., Bowler-Barnett, E. H., Britto, R., Bye-A-Jee, H., Cukura, A., Denny, P., Dogan, T., Ebenezer, T. G., Fan, J., Garmiri, P., da Costa Gonzales, L. J., Hatton-Ellis, E., Hussein, A., Ignatchenko, A., … Zhang, J. (2023). UniProt: the Universal Protein Knowledgebase in 2023. *Nucleic Acids Research*, *51*(D1). https://doi.org/10.1093/nar/gkac1052

Bird, S., Klein, E., & Loper, E. (2016). NLTK Book. In *O'Reilly*.

Davis, A. P., Wiegers, T. C., Johnson, R. J., Sciaky, D., Wiegers, J., & Mattingly, C. J. (2023). Comparative Toxicogenomics Database (CTD): update 2023. *Nucleic Acids Research*, *51*(D1). https://doi.org/10.1093/nar/gkac833

Lee, Y., Son, J., & Song, M. (2022). BertSRC: transformer-based semantic relation classification. *BMC Medical Informatics and Decision Making*, *22*(1). https://doi.org/10.1186/s12911-022-01977-5

Lipscomb, C. E. (2000). Medical Subject Headings (MeSH). In *Bulletin of the Medical Library Association* (Vol. 88, Issue 3).

Mikolov, T., Chen, K., Corrado, G., & Dean, J. (2013). [Word2Vec] Distributed Representations of Words and Phrases and their Compositionality (Google 2013). *CrossRef Listing of Deleted DOIs*, *1*.

*PubMed Index of daily update files*. (2024). https://ftp.ncbi.nlm.nih.gov/pubmed/updatefiles/

Saneesh Mohammed, N., & Nazeer, K. A. A. (2013). An improved method for extracting acronym-definition pairs from biomedical Literature. *2013 International Conference on Control Communication and Computing, ICCC 2013*. https://doi.org/10.1109/ICCC.2013.6731649

Sayers, E. W., Bolton, E. E., Brister, J. R., Canese, K., Chan, J., Comeau, D. C., Connor, R., Funk, K., Kelly, C., Kim, S., Madej, T., Marchler-Bauer, A., Lanczycki, C., Lathrop, S., Lu, Z., Thibaud-Nissen, F., Murphy, T., Phan, L., Skripchenko, Y., … Sherry, S. T. (2022). Database resources of the national center for biotechnology information. *Nucleic Acids Research*, *50*(D1). https://doi.org/10.1093/nar/gkab1112

Schwartz, A. S., & Hearst, M. A. (2003). A simple algorithm for identifying abbreviation definitions in biomedical text. *Pacific Symposium on Biocomputing. Pacific Symposium on Biocomputing*. https://doi.org/10.1142/9789812776303_0042

Seal, R. L., Braschi, B., Gray, K., Jones, T. E. M., Tweedie, S., Haim-Vilmovsky, L., & Bruford, E. A. (2023). Genenames.org: the HGNC resources in 2023. *Nucleic Acids Research*, *51*(D1). https://doi.org/10.1093/nar/gkac888

Zdrazil, B., Felix, E., Hunter, F., Manners, E. J., Blackshaw, J., Corbett, S., de Veij, M., Ioannidis, H., Lopez, D. M., Mosquera, J. F., Magarinos, M. P., Bosc, N., Arcila, R., Kizilören, T., Gaulton, A., Bento, A. P., Adasme, M. F., Monecke, P., Landrum, G. A., & Leach, A. R. (2023). The ChEMBL Database in 2023: a drug discovery platform spanning multiple bioactivity data types and time periods. *Nucleic Acids Research*. https://doi.org/10.1093/nar/gkad1004
